# Time-Critical Emergencies in Suburban Parks and Outdoor Recreation Spaces

**DOI:** 10.7759/cureus.75481

**Published:** 2024-12-10

**Authors:** Alexandra Vaughn, Matthew J Levy, Jenna Paul-Schultz, Lia Melvin, Becca Scharf, Asa Margolis, J. Lee Jenkins

**Affiliations:** 1 Department of Emergency Medicine, Johns Hopkins University School of Medicine, Baltimore, USA; 2 Department of Fire and Rescue Services, Howard County Maryland, Marriottsville, USA

**Keywords:** emergencies in outdoor recreational locations, ems activations in parks, outdoor activities, prehospital ems, time critical outdoor emergencies

## Abstract

Objective

Prior studies have described the patterns of emergency medical service (EMS) activations in national parks in the United States. However, little data exists regarding EMS activations in local and regional outdoor recreational locations. We performed a retrospective analysis of EMS activations originating from parks and recreational areas in suburban Howard County, Maryland, to characterize those activations determined to be time-critical emergencies.

Methods

EMS activations originating from outdoor recreational areas and parks in Howard County, Maryland, between December 2018 and December 2022, were extracted from the Howard County Department of Fire and Rescue Services (HCDFRS) electronic medical records database. Patient complaints were categorized into medical or traumatic etiologies and then determined to be “time-critical” or “non-time-critical” by two emergency physician reviewers with a tie-breaker reviewer to resolve discrepancies.

Results

A total of 396 EMS activations in county parks and recreational areas were recorded during the study period, with 61 (15.40%) determined to be time-critical. The most commonly occurring time-critical calls were cardiac arrest (7; 11.48%), drug/alcohol-related complaints (5; 8.20%), and altered mental status (5; 4.92%). Most EMS activations were for male patients (273; 68.94%). Pediatric patients (<18 years old) constituted 161 (40.66%) time-critical traumatic injury activations, and these numbers decreased as patient age increased. The most commonly occurring time-critical medical activations (11; 2.78%), occurred in 40-64-year-olds. The highest number of total EMS activations occurred in October, with 57 calls (14.39%), and the greatest volume of time-critical activations occurred during June, with nine calls (14.75%).

Conclusion

Within a suburban county’s EMS system, 15.4% of the total EMS activations to parks and outdoor recreational locations were found to be time-critical, significantly higher than in other places. Patients aged 0-18 experienced the highest number of time-critical trauma-related activations, whereas those aged 40-64 had the highest number of time-critical medical activations. EMS agencies should review their activation rates in these locations to determine the need to enhance education and operational readiness for response to time-critical activations for pediatric trauma and adult medical emergencies.

## Introduction

Outdoor recreational activities continue to grow in popularity in the United States (US). A national survey performed in 2003 found that approximately 97.6% of U.S. citizens aged 16 and older participated in outdoor recreational activities in the preceding year [1,2]. In 2023, about 325 million people visited US National Parks [3]. Prior research has documented injury and illness patterns in multiple national parks, including Shenandoah and Yellowstone National Parks [1,4]. A previous analysis of the US National Parks reported an incidence of EMS activations of 45.9 events per 1 million visitors [5,6]. Also, within the US National Park Service, there was an average of 8 deaths per 10 million visits to park sites between 2007 and 2018, with the leading causes being drownings, motor vehicle accidents, and falls [7,8]. Beyond its vast and expansive system of national parks, the US is also home to over 10,000 local and regional parks and outdoor recreation spaces, including playgrounds, sports fields, and hiking trails, which receive more than 1.1 billion visits annually [9]. However, little data exists regarding EMS activations and responses to these locations.

Maryland hosts 65 state parks, which saw an increase in visitation from 14.9 million in 2019 to 20.6 million in 2021 [10]. Howard County, Maryland’s sixth largest county, is located in the geographic center of Maryland. Howard County has 43 state, regional, and neighborhood parks and natural resource areas encompassing 9,768 acres across approximately 254 square miles and a population of 335,000 [11-13]. We aimed to characterize EMS activations in outdoor recreational spaces in Howard County, specifically describing those time-critical emergency conditions that require intervention within minutes to save a life or maintain essential functions [14,15].

## Materials and methods

This was a retrospective, multi-year analysis of EMS activations in parks and outdoor recreation spaces in Howard County, Maryland, between December 2018 and December 2022. Howard County is a suburban county between Baltimore and Washington, DC, with a population of 335,000 people. The Howard County Department of Fire and Rescue Services (HCDFRS) is the sole agency responsible for EMS services in Howard County and responds to approximately 30,000 calls for service annually. The department's prehospital electronic medical records database (ImageTrend Elite, Lakeville, MN, US) was queried for all calls encoded at a park or outdoor recreational space. All EMS activations that generated a response for HCDFRS to a park or outdoor recreation space were included. Activations were excluded if the call was canceled before EMS arrival or patient contact was not made. Scene and incident-related variables were extracted, and the patient’s chief complaint was categorized as either a medical or traumatic complaint, as well as the anatomical location and the mechanism of injury.

Each case, including patient care narratives, was screened by two independent emergency physicians (ACV and JPS) to determine if the case was a time-critical activation, as previously defined by several study investigators (MJL, AMM, et al.) [15]. This definition was based upon a prehospital intervention required in minutes to save life, maintain essential functions, or a diagnosis/impression associated with an illness or injury with a known time-critical intervention that impacts outcome (Table 1), and further adapted by the study team to align with to Maryland’s EMS protocols [16]. A third reviewer (MJL) served as a tiebreaker. Data were reviewed and analyzed using Microsoft Excel (Microsoft Corporation, Redmond, WA, US), and descriptive and summary statistics were performed. This study was reviewed and approved by the Johns Hopkins Institutional Review Board.

**Table 1:** Time-critical EMS interventions and conditions EMS: emergency medical service

Time Critical Interventions	Time Critical Conditions
AED Defibrillation	Acute Bronchospasm
AICD/Pacer Deactivation Magnet	Acute Respiratory Distress (Dyspnea)
Amiodarone	Allergic Reaction
Atropine	Altered Mental Status
Back Blows	Amputation of Limb
Bleeding Control	Anaphylaxis
Calcium Chloride	Asthma
Calcium Gluconate	Carbon Monoxide Poisoning
Cardioversion	Cardiac Arrest
Chest Seal	Cardiac Arrhythmia/Dysrhythmia
Chest Thrusts	Cardiogenic Shock
CPAP	Chest Pain / Discomfort
CPR	Congestive heart failure (CHF)
Dopamine	Convulsions
Droperidol	Diabetic Hypoglycemia
DuoDote	Drowning
Epinephrine	Electrocution
Etomidate	Foreign Body in Respiratory Tract
Haloperidol	Heatstroke and Sunstroke
Heimlich Maneuver	Hemorrhage
Hemostatic Agent	Hemorrhagic Shock
Hydroxocobalamin	Hypoglycemia (Not Diabetic)
Intraosseous	Hypotension
Ketamine	Hypothermia
Magill Forceps	Hypovolemia/Shock
Magnesium Sulfate	Inhalation Injury (Toxic Gas)
Manual Defibrillation	Labor and Delivery Complications
Mechanical CPR	Neurogenic Shock
Midazolam	Obstetric Trauma
Naloxone	Overdose - Amphetamine
Needle Cricothyroidotomy	Overdose - Cocaine
Nasal Airway	Overdose - Heroin
Oral Airway	Overdose - Methadone
Pacing	Overdose - Opium
Pelvic Binder	Overdose - Other Opioids
Pleural Decompression	Poisoning / Drug Ingestion
STEMI Alert	Postpartum Hemorrhage
Stroke Alert	Preterm Labor With Preterm Delivery
Succinylcholine	Pulmonary Edema, Acute
Suction	Respiratory Arrest
Surgical Cricothyroidotomy	Respiratory Condition Due to Chemicals, Gases, Fumes, &Vapors
Tourniquet	Respiratory Distress of Newborn
Traction Splint	Respiratory Failure
Tracheal Intubation	Seizures With Status Epilepticus
Tranexamic Acid (TXA)	Septic Shock
Vecuronium	ST-Elevation Myocardial Infarction (STEMI)
	Stroke
	Suffocation or Asphyxia
	Tracheostomy Problem
	Transient Cerebral Ischemic Attack (TIA)
	Traumatic Circulatory Arrest

## Results

**Figure 1:**
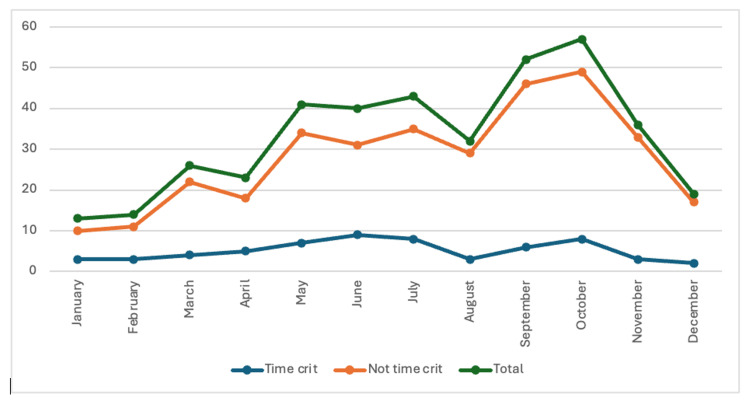
EMS activations by month in parks and recreational areas (n=396) EMS: emergency medical service

During the four-year study period, 396 EMS activations occurred in parks and recreational areas in Howard County. The most calls occurred in 2019 and 2021, totaling 115 calls each, and the lowest number of activations (66) occurred in 2020, coinciding with the COVID-19 pandemic. Across all years, October had the highest number of EMS activations (57, 14.39%) while January had the fewest activations (13; 3.28%) (Figure 1).

Most EMS activations originated from athletic fields (163; 41.16%), followed by forest/trail/wilderness locations (100; 25.25%). Male patients comprised the greatest proportion of the total activations (273; 68.94%) and had the highest number of overall total traumatic activations (197; 49.75%) and traumatic time-critical activations (26; 83.87%). Similarly, males experienced more overall total medical activations (69; 17.42%) and medical time-critical activations (23; 79.31%) than female patients. Patients 0-18 comprised the largest proportion of activations by age group, accounting for 161 (40.66%) activations, and had the highest number of time-critical traumatic activations of any age group (13; 3.28%). Table 2 further displays population-related and demographic breakdowns.

**Table 2:** Demographics of EMS activations in parks and recreational areas (n = 396) EMS: emergency medical service

	Trauma time-critical (%)	Trauma non-time critical (%)	Medical time-critical (%)	Medical non-time critical (%)	Total
Age					
0-18	13 (42%)	122 (49%)	5 (17%)	20 (25%)	160 (41%)
19-39	8 (26%)	58 (23%)	9 (31%)	21 (26%)	96 (25%)
40-64	9 (29%)	37 (15%)	11 (38%)	24 (30%)	81 (21%)
65+	1 (3%)	30 (12%)	4 (14%)	12 (15%)	47 (12%)
Unknown	–	–	–	3 (4%)	3 (1%)
Sex					
Female	5 (16%)	76 (31%)	6 (21%)	32 (40%)	121 (31%)
Male	26 (84%)	171 (69%)	23 (79%)	46 (58%)	273 (69%)
Unknown	–	–	–	2 (3%)	2 (1%)
Race					
White	19 (61%)	140 (57%)	11 (38%)	34 (43%)	204 (53%)
Black/African American	3 (10%)	50 (20%)	9 (31%)	24 (30%)	86 (22%)
Hispanic or Latino	3 (10%)	23 (9%)	1 (3%)	5 (6%)	32 (8%)
Native Hawaiian or Other Pacific Islander	1 (3%)	2 (1%)	0 (0%)	0 (0%)	3 (1%)
Asian	1 (3%)	7 (3%)	3 (10%)	4 (5%)	15 (4%)
Other	4 (13%)	25 (10%)	5 (17%)	13 (16%)	47 (12%)

Traumatic injuries were the most common reason for EMS activations, accounting for 278 (70.20%) of all activations. Among patients with traumatic injuries, 256 (89.51%) were from blunt injuries and 175 (58.33%) were from sports-related trauma. Lower extremity injuries were the most common of total injuries (106; 36.93%), followed by both upper extremity and head injuries, each accounting for 61 (21.25%) of the total traumatic injuries. Overall, 61 (15.40%) of all EMS activations were determined to be time-critical. Both traumatic and medical time-critical activations were more evenly divided, with 31 (50.82%) and 29 (47.54%) activations, respectively. One patient had evidence of both medical and traumatic etiologies. Time-critical medical activations occurred most frequently in patients 40-64 years (11; 2.78% of total calls).

Cardiac arrest was the most common medical time-critical complaint (7; 11.48%), followed by drug and alcohol-related complaints (5; 8.20%), and altered mental status (5; 4.92%). The largest proportion of time-critical trauma activations was for sports-related trauma (10; 31.25%), bicycle-related trauma (7; (21.88%), and falls from a height (5; 15.63%). Tables 3-4 describe the top six traumatic and medical time-critical etiologies. The largest number of time-critical EMS activations (n = 9, 14.75%) occurred in June, while the smallest (n = 2, 3.28%) occurred in December. Most time-critical activations originated from forest/trails/wilderness areas (24; 39.34%), followed by athletic fields (23; 37.70%) and athletic courts (11; 18.03%). Table 5 further details the location of time-critical EMS activations.

**Table 3:** Most common time-critical traumatic conditions (total time-critical traumatic EMS activations; n=31) EMS: emergency medical service

Etiology	n (%)
Sports-related trauma	10 (31.25%)
Bicycle-related trauma	7 (21.88%)
Fall from height	5 (15.63%)
Fall from seated/standing	3 (9.38%)
Motor vehicle-related trauma	3 (9.38%)
Blunt object, accident	2 (6.25%)

**Table 4:** Most common time-critical medical conditions (total time-critical medical EMS activations; n=29) EMS: emergency medical service; ETOH: ethyl alcohol

Etiology	n (%)
Cardiac Arrest	7 (11.48%)
Drug/ETOH related	5 (8.20%)
Altered Level of Consciousness/Mental Status	3 (4.92%)
Pain (General)	3 (4.92%)
Seizure (Active or Resolved)	3 (4.92%)
Allergic Reaction	2 (3.28%)

**Table 5:** Locations of time-critical EMS activations (total time-critical activations; n=61) EMS: emergency medical service

Incident Location	n (%)
Forest/Trail/Wilderness – Recreation	24 (39.34%)
Athletic Field (Grass/Dirt Surface) – Recreation	23 (37.70%)
Athletic Court (Hard Surface) – Recreation	11 (18.03%)
Playground – Recreation	1 (1.64%)
Water Related (River/Lake/Pond)	1 (1.64%)
Other – Recreation	1 (1.64%)

## Discussion

Emergency medical services systems must be ready to respond to time-critical emergencies, including those occurring in potentially challenging and unique situations. Outdoor green spaces are no exception. These locations are essential for a community’s well-being, provide diverse recreational opportunities, and are a significant economic driver [17,18]. Previous studies examining prehospital activations in wilderness settings across the United States have highlighted patient demographics, injury mechanisms, illness trends, seasonal variability, required interventions, and EMS transportation methods [1,3,4,6,7,10]. Although some studies have reported the level of care administered, none have specifically stratified activations based on the time-criticality of the presenting condition, making this study unique. Given the ever-increasing strain and demand on EMS resources, prioritizing readiness efforts based on time-critical conditions is essential for optimal patient outcomes. This analysis found a 15.4% rate of time-critical conditions in parks and outdoor recreation locations. This rate is more than twice that reported in a multi-center review of approximately 1.7 million activations, of which 6% were time-critical (*p *=<0.05) [15].

The largest number of time-critical calls occurred in June, whereas the most activations occurred in October. This may be correlated with the location, as most time-critical activations originated from forests/trails/wilderness recreation settings, which may see a higher volume of visitation during the summer months. In contrast, most non-time-critical activations originated from athletic fields, with many organized sports occurring in the autumn months. A bimodal distribution of age was observed, with most time-critical traumatic activations occurring in those between 0 and 18 years old, whereas most time-critical medical activations occurred in those between 40 and 64 years old. Regarding gender, our study followed patterns found in Stephens et al., with most activations being for male patients, who also had the highest time-critical traumatic activations and time-critical medical activations [19]. Johnson et al. and Baker et al. reported a more even distribution between male and female patients [1,4]. Most time-critical medical incidents were for cardiac arrest, followed by drug/alcohol-related complaints, followed by altered mental status. Unlike Baker et al., who found most overall medical activations to be for complaints of dizziness, or Leemon et al., who found vomiting/diarrhea to be the primary medical complaint, our leading medical activation was for generalized pain with syncope/dizziness having the next highest incidence, followed by drug/alcohol related complaints [4,20]. This information can help local officials and leaders as they seek ways to implement programs to make parks and recreational spaces safer. For example, Howard County recently added 17 publically located automated external defibrillators in weatherproof enclosures throughout Howard County's parks [21].

This study has several limitations. This analysis occurred in one county, and the results may not be generalizable to other locations. The number of annual visitors to Howard County parks and recreational spaces is unknown, making calculating comparative rates of illness and injuries difficult. We acknowledge this lack of robust visitation data as a limitation. The authors also acknowledge many differences between EMS activations and responses within local and regional parks compared to national parks. This study was conducted with a single EMS system, which introduces a notable level of bias. Potential bias also exists from reliance on EMS documentation for data abstraction. We additionally recognize the potential for survivorship bias in the population served by the EMS system since this analysis is limited to cases where someone called 911 and the patient was also present to receive care from the responders. As a retrospective analysis, these findings should be interpreted as hypothesis-generating rather than hypothesis-testing, and these results may not be generalizable to every EMS system.

## Conclusions

In suburban Howard County, Maryland’s EMS system, 15.4% of all EMS activations to parks and outdoor recreational locations were identified as time-critical and at a higher rate than in other places compared to a recent large dataset. There was an equal distribution of medical and traumatic etiologies among these time-critical emergencies. The most significant number of time-critical trauma-related activations occurred in patients aged 0-18 while the most significant number of time-critical medical activations occurred in patients aged 40-64. These findings suggest that EMS agencies should review their activation and patient presentation rates in their response areas. This review could help local officials and EMS leaders develop and implement strategies to improve the prevention, response, and mitigation of emergency medical situations in outdoor recreational areas. Further studies are needed to assess EMS activation patterns, patient presentations, and outcomes across greater regions and better characterize EMS encounters in these unique environments.
